# The Effect of Bariatric Surgery on Circulating Levels of Monocyte Chemoattractant Protein-1: A Systematic Review and Meta-Analysis

**DOI:** 10.3390/jcm11237021

**Published:** 2022-11-28

**Authors:** Tannaz Jamialahmadi, Mitra Abbasifard, Željko Reiner, Prashant Kesharwani, Amirhossein Sahebkar

**Affiliations:** 1 Applied Biomedical Research Center, Mashhad University of Medical Sciences, Mashhad, Iran; 2 Surgical Oncology Research Center, Mashhad University of Medical Sciences, Mashhad, Iran; 3 Immunology of Infectious Diseases Research Center, Research Institute of Basic Medical Sciences, Rafsanjan University of Medical Sciences, Rafsanjan, Iran; 4 Department of Internal Medicine, Ali-Ibn Abi-Talib Hospital, School of Medicine, Rafsanjan University of Medical Sciences, Rafsanjan, Iran; 5 Department of Internal Medicine, University Hospital Center Zagreb, 10000 Zagreb, Croatia; 6 Department of Pharmaceutics, School of Pharmaceutical Education and Research, Jamia Hamdard, New Delhi 110062, India; 7 Center for Transdisciplinary Research, Department of Pharmacology, Saveetha Dental College, Saveetha Institute of Medical and Technical Science, Chennai 602105, India; 8 Biotechnology Research Center, Pharmaceutical Technology Institute, Mashhad University of Medical Sciences, Mashhad, Iran; 9 Department of Biotechnology, School of Pharmacy, Mashhad University of Medical Sciences, Mashhad, Iran

**Keywords:** obesity, bariatric surgery, monocyte chemoattractant protein-1, atherosclerosis, meta-analysis, cardiovascular disease

## Abstract

**Background**: MCP-1 (monocyte chemoattractant protein) plays an important role in early phases of atherogenesis as well as in plaque destabilization, which causes cardiovascular events to play an important role in low-grade inflammation. Obesity, particularly extreme obesity, is a pivotal risk factor for atherosclerosis and many other diseases. In the early stages, bariatric surgery might stop or slow atherogenesis by suppressing inflammation, but also in later stages, preventing plaque destabilization. The aim of this meta-analysis was to provide an answer as to whether bariatric surgery has a significant effect on circulating MCP-1 level or not. **Methods**: A systematic literature search in PubMed, Scopus, Embase, and Web of Science was performed from inception to 1 January 2022. Meta-analysis was performed using Comprehensive Meta-Analysis (CMA) V2 software. In order to heterogeneity compensation of studies in terms of study design and treatment duration, the characteristics of the studied populations random-effects model and the generic inverse variance weighting method were used. To investigate the relationship with the estimated effect size, a random-effect meta-regression model was used. To assess the exitance of publication bias in the meta-analysis, the funnel plot, Begg’s rank correlation, and Egger’s weighted regression tests were used. **Results**: Meta-analysis of 25 studies with 927 subjects included demonstrated a significant decrease of MCP-1 concentration after bariatric surgery. The data of meta-regression did not indicate any association between the alterations in body mass index (BMI) and absolute difference in MCP-1 levels, but a linear relationship between the changes in MCP-1 and length of follow-up was proven. **Conclusions**: Bariatric surgery significantly decreases MCP-1 concentration, but there was no association between the changes in BMI and absolute difference in MCP-1 levels before and after the surgery.

## 1. Introduction

Obesity causes low-grade chronic inflammation, which is marked by abnormal cytokine production, increased synthesis of acute-phase reactants, and activation of pro-inflammatory signaling pathways [1]. In the adipose tissue of obese patients, the accumulation of macrophages causes macrophage-elicited inflammation and adipocyte-macrophage interaction, which are important processes in obesity. They occur due to hypertrophic adipocyte-derived MCP-1)/C-C chemokine receptor 2 (CCR2) pathway and participate in a vicious cycle that aggravates inflammation in the adipose tissue [2]. This is important, since it has to be stressed again that low grade inflammation is one of the main characteristics of atherogenesis.

It has been shown that MCP-1 (monocyte chemoattractant protein)—as a member of the CC chemokine subfamily—recruits immune cells to the peripheral tissues during inflammation. It plays a pivotal role in atherogenesis as well, particularly in the early phases of atherogenesis, since atherogenesis is also an inflammatory condition. Monocytes are recruited to the arterial wall by MCP-1 and experimental studies, suggested that inhibiting MCP-1 signaling could slow down atherosclerosis progression and atherosclerotic plaque destabilization, which causes cardiovascular events [3,4].

Bariatric surgery is a surgical treatment primarily for obese patients, which improves metabolic and inflammatory processes as well as cardiometabolic risk factors beyond weight loss [5,6,7,8,9,10,11,12,13,14,15]. The types of bariatric surgery are sleeve gastrectomy (SG), laparoscopic adjustable gastric band (LAGB), Roux-en-Y gastric bypass (RYGP), biliopancreatic diversion/duodenal switch (BPD/DS), and one anastomosis gastric bypass/mini gastric bypass (OAGB/MGB) [16]. In the early stages, bariatric surgery might prevent or slow atherogenesis by breaking the vicious circle between endothelial dysfunction and inflammation, but also in later stages, preventing plaque destabilization [17].

Despite many studies, there is still no clear answer whether bariatric surgery has a significant effect on circulating MCP-1 level or not. Therefore, the aim of this systematic review and meta-analysis was to provide the answer to this question.

## 2. Methods

### 2.1. Search Strategy

The 2009 preferred reporting items for systematic reviews and meta-analysis (PRISMA) guidelines were used to make this systematic review and meta-analysis [18]. From inception to 1 January 2022, Scopus, PubMed, Embase, Google scholar, and Web of Science were searched using the following keywords in titles and abstracts (including when used MESH terms): (“bariatric surgery” OR gastrectom* OR gastroplast* OR “Roux-en-Y” OR “gastric bypass” OR “biliopancreatic diversion” OR “duodenal switch” OR “gastrointestinal diversion” OR “weight loss surgery” OR gastroenterostom* OR “jejunoileal bypass” OR “obesity surgery” OR “weight-loss surgery” OR “sleeve surgery” OR “bariatric procedure” OR “metabolic surgery” OR “gastric band”) AND (“monocyte chemoattractant protein-1” OR “MCP-1” OR MCP1 OR “MCP 1”).

### 2.2. Study Selection

The eligibility criterion of the included studies was only peer-reviewed original publications written in English which reported MCP-1 concentration before and following bariatric surgery. All animal studies, abstract-only publications, non-English papers, duplicate research, reviews, case reports, meta-analyses, comments, letters, and studies without outcomes, and those with no surgical intervention were excluded.

### 2.3. Data Extraction

The titles and abstracts of the included publications were checked by two blinded authors independently (TJ and AS). The full texts of the chosen papers were gathered for a second review. In the case of the same organization and/or authors in same study, the larger study concerning the sample size was included. Any disagreement was reconciled with consensus and discussion. The extraction of following data was done: the identity of the first author and the design of the study, the year of publication, the type of surgery and length of follow-up, patients characteristics, and clinical outcomes.

Primary outcome: the effect of bariatric surgery on MCP-1 concentration.

Secondary outcome: the effect of body mass index (BMI) changes and length of follow-up on MCP-1 levels.

### 2.4. Quality Assessment

The Newcastle–Ottawa Scale was used to assess the quality of eligible studies. The scale is divided into three broad stratifications: selection (consists of four items), confounder (including one item), and exposure (contains two items), each with a maximum score of four, one, and two points [19,20].

### 2.5. Quantitative Data Synthesis

A meta-analysis was performed using comprehensive meta-analysis (CMA) V2 software [21]. For continuous outcomes, the weighted mean difference (WMD) with associated confidence intervals was presented. To calculate WMD means, standard deviations (SD) and sample sizes were needed. The mean and standard deviation values were calculated by the method described previously if the outcome measures were reported in median and interquartile range (or 95% confidence intervals [CI]). SD was determined using this formula: SD = SEM × sqrt (number of participants). Additionally, pooled standard deviation was used to deal with missing SD. The overall estimate of effect size was calculated using a random effects meta-analysis. A random-effects model (using DerSimonian-Laird method) and the general inverse variance weighting technique were employed to account for heterogeneity of publications in terms of study design, features of the populations and treatment duration [18]. To examine the effect of each study on the overall effect size, we conducted a sensitivity analysis using the leave-one-out strategy (i.e., exclusion of one study at a time to evaluate its impact on the overall result) [22].

### 2.6. Meta-Regression

BMI change before and after the surgery, as well as follow-up duration, were the independent variables in a random-effect meta-regression model to explore the effect of these variables on effect size.

### 2.7. Subgroup Analysis

We classified the publications based on follow-up duration to illustrate the source of heterogeneity into <12 months and ≥12 months. Another sub-analysis was also performed, taking into consideration the two most prevalent types of surgery (LSG and RYGB).

### 2.8. Publication Bias

The “trim and fill” test was used to adjust the results when the funnel plot initially showed asymmetry. Then, Egger’s and Begg’s tests were applied to statistically evaluate publication bias. When a significant result occurred, the number of potentially missing studies required to make the *p*-value non-significant was calculated using the “fail-safe N” approach [23].

## 3. Results

**Figure 1:**
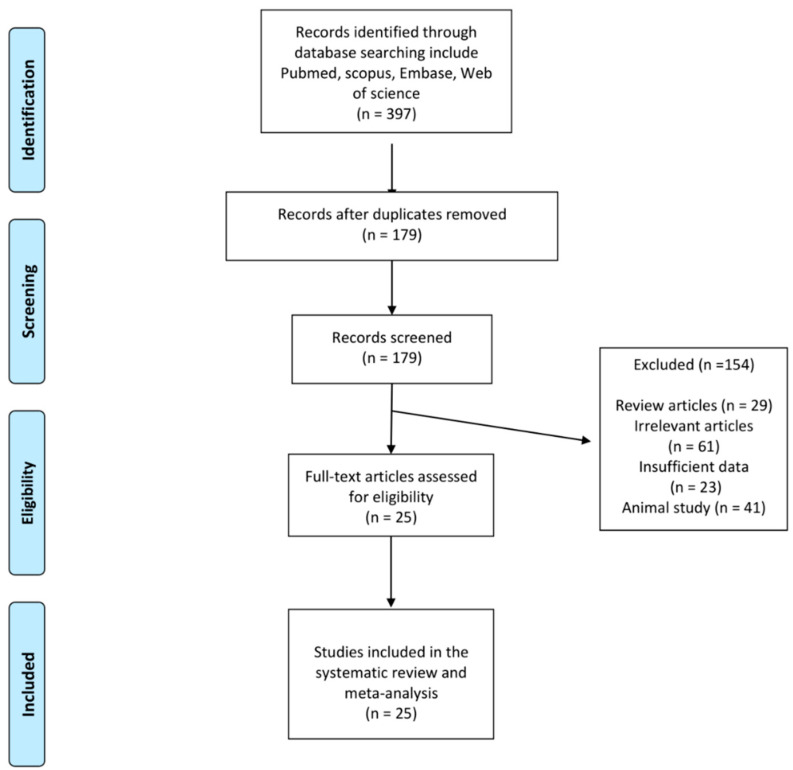
Flow chart of identified publications and those included into meta-analysis.

The database search yielded 397 publications, 179 of which remained after exclusion of duplications. Overall, 154 studies were not included (29 publications were reviews, 61 publications were excluded for not fulfilling the inclusion criteria, 23 studies did not report enough data, and 41 were animal studies). As a result, 25 studies measuring circulating MCP-1 following bariatric surgery were analyzed (Table 1). The study selection procedure is presented in Figure 1.

### 3.1. Quality Assessment of the Included Studies

Among 24 nonrandomized studies, all of the selected publications represented the exposed cohort, and ascertainment of exposure. All of them demonstrated that the outcome of interest was not present at the start of the study. Eventually, most of considered publications met the ascertainment of outcome criteria. Cochrane Collaboration’s tool assessed the risk of bias in one randomized study. The quality of the included publications is assessed in Table 2.

### 3.2. Primary Outcome

#### Effect of Bariatric Surgery on MCP-1 Concentration

**Figure 2:**
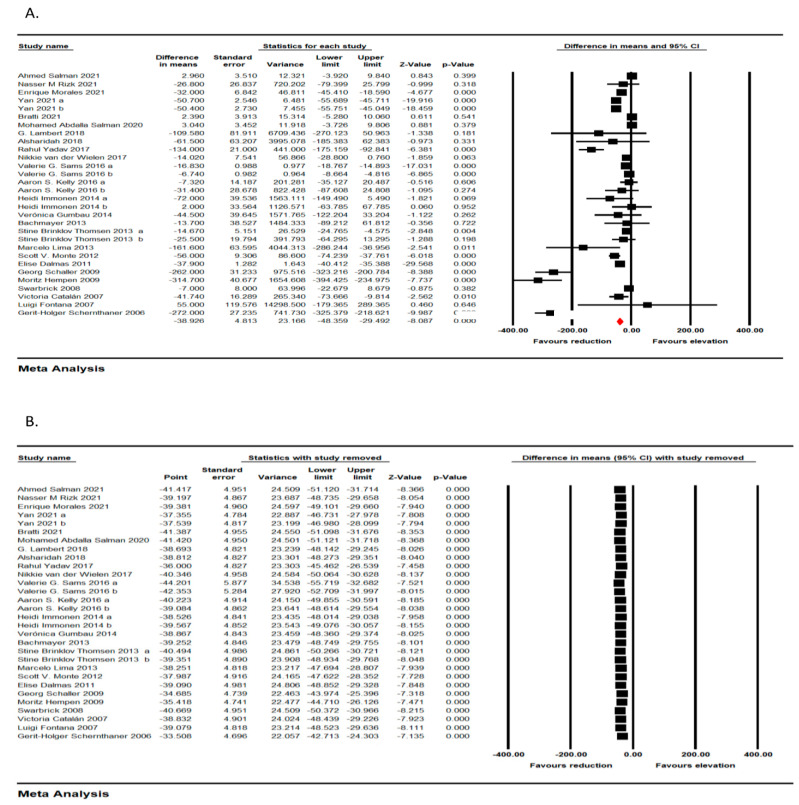
(**A**) Forest plots representing standardized mean difference and 95% confidence intervals (CIs) for the effect of bariatric surgery on MCP-1; (**B**) Leave-one-out sensitivity analysis for the effect of bariatric surgery on MCP-1.

A total of 25 trials, including 927 individuals, confirmed a significant reduction in MCP following bariatric surgery (WMD: −38.926, 95% CI: −48.359, −29.492, *p* < 0.001) (Figure 2A). The reduction of MCP-1 concentration was robust in the leave-one-out sensitivity analysis (Figure 2B).

### 3.3. Secondary Outcomes

#### Meta-Regression

**Figure 3:**
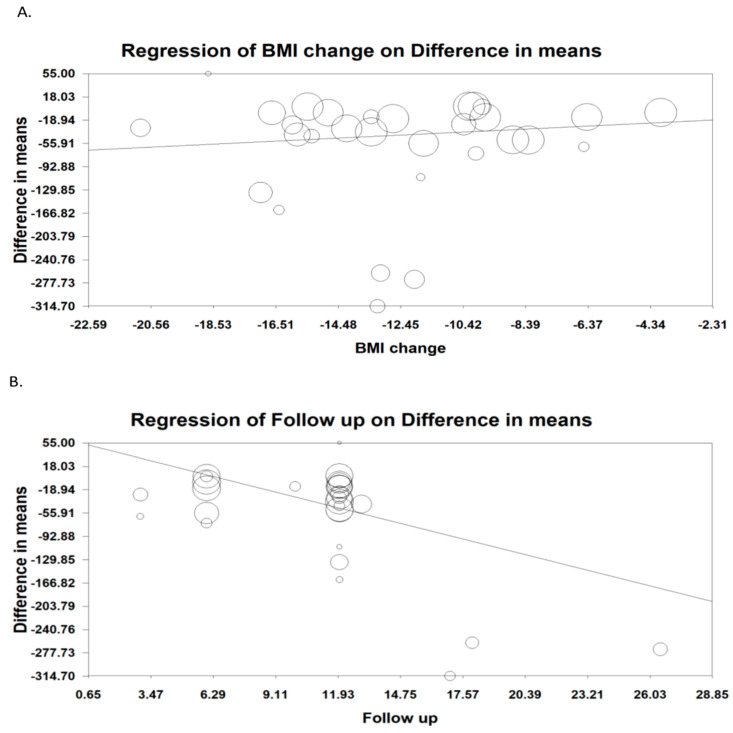
Random effect meta-regression for evaluating the effect of: (**A**) BMI change; (**B**) Follow-up duration.

The results of meta-regression, which were used to assess the effect of various variables on the reduction of post-surgery circulating MCP-1, did not show any association between the changes in body mass index (BMI) and absolute difference in MCP-1 levels (slope: 2.378; 95% CI: 0.470, 5.226; *p* = 0.101). The results showed a linear relationship between the changes in MCP-1 and length of follow-up (slope: −8.814; 95% CI: −11.068, −6.559; *p* < 0.001) (Figure 3A,B).

### 3.4. Subgroup Analyses

**Figure 4:**
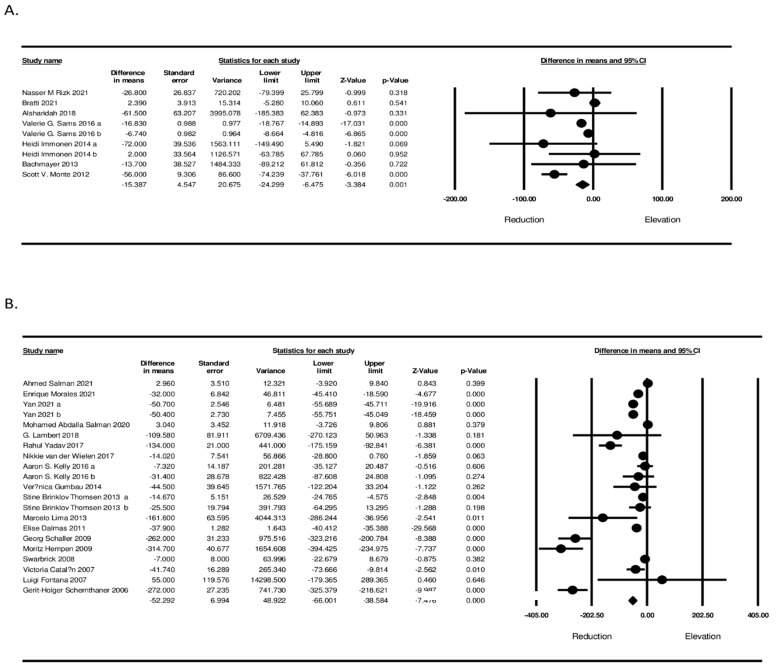
Subgroup analysis based on follow-up duration ((**A**), less than 12 months), ((**B**), equal or more than 12 months).

**Figure 5:**
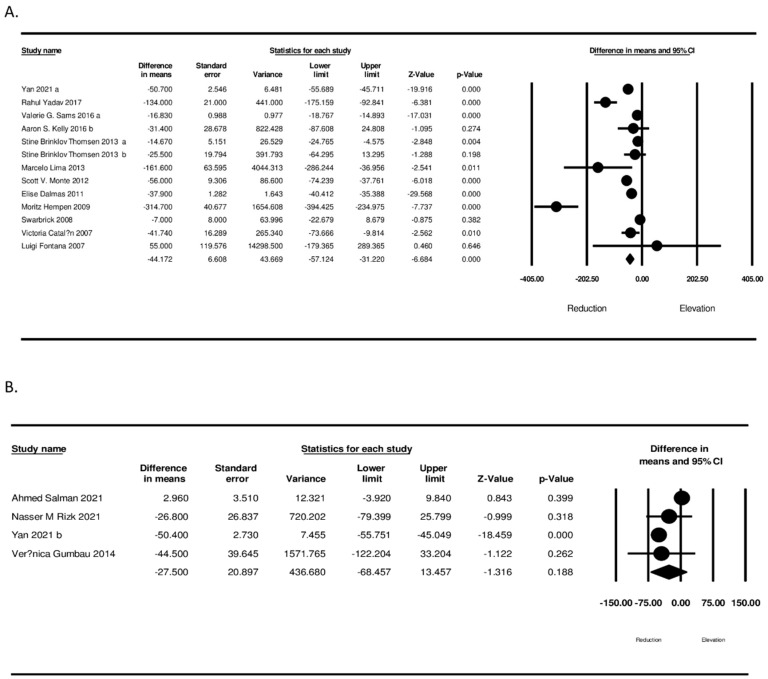
Subgroup analysis based on type of surgery ((**A**) RYGB) ((**B**) LSG).

In the sub-analyses, a significant difference in changes of circulating MCP-1 based on the length of follow-up (≥12 months and <12 months) (WMD: −15.387, 95% CI: −24.299, 9.620, *p* < 0.001; I^2^: 96.87 for <12 months and WMD: −26.350, 95% CI: −33.822, −18.878, *p* < 0.001; I^2^: 89.43 for ≥12 months) was shown (Figure 4). Furthermore, according to the type of bariatric surgery, there was a significant reduction in circulating MCP-1 concerning the type of bariatric surgery (WMD: −27.500, 95% CI: −68.457, 13.457, *p* < 0.001; I^2^: 97.91 for LSG and WMD: −44.172, 95% CI: −57.124, −31.220, *p* < 0.001; I^2^: 96.74 for RYGB) (Figure 5).

### 3.5. Publication Bias

**Figure 6:**
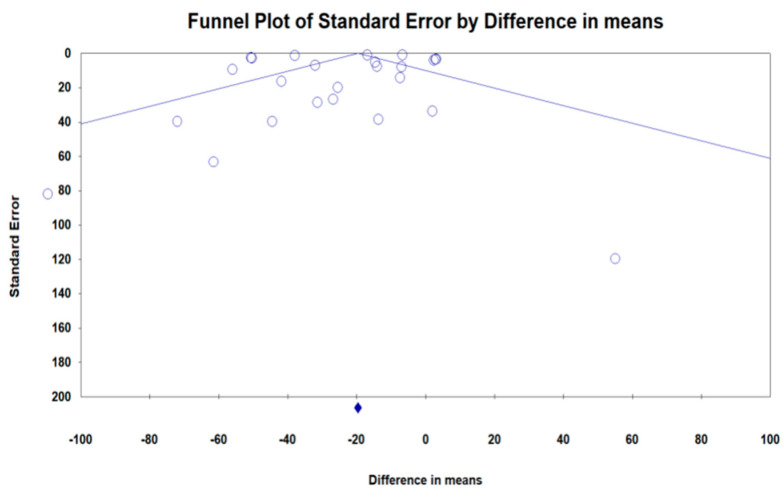
Funnel plot detailing publication bias in the publications describing the effect of BS on MCP-1.

As shown in Figure 6, funnel plot asymmetry test assessed the publication bias of the studies.

Publication bias did not exist based on Egger’s (intercept = −2.02, standard error = 1.295; 95% CI = −4.682, 0.623, t = 1.567, df = 28, two-tailed *p* = 0.128) and Begg’s tests (Kendall’s Tau with continuity correction = −0.193, z = 1.498, two-tailed *p*-value = 0.133) in detecting the impact of bariatric surgery on circulating MCP-1. Trim and fill test showed one “missing” study in order to adjust publication bias. Furthermore, “fail-safe N” analysis showed that 6014 papers could change the conclusions of this study (Figure 5).

## 4. Discussion

The results of this meta-analysis showed a significant decrease of MCP-1 concentration after bariatric surgery. It is important to stress that there was no association between the changes in BMI and absolute difference in MCP-1 levels, but a linear relationship between the changes in MCP-1 and the length of follow-up was shown.

MCP-1 is important in the atherogenesis and destabilization of atherosclerotic plaques, particularly in the early stages of atherogenesis. As a non-traditional diagnostic marker for atherosclerosis, high levels of MCP-1 may contribute to low-grade inflammation in obesity [47,48].

In an earlier study, one year following bariatric surgery, there was a considerable decrease in cytokines such as MCP-1. Weight loss improved adiposity serum biomarkers and obesity-related comorbidities [49]. Christiansen et al. [50] investigated a reduction in MCP-1 concentration after weight loss in severe obesity, and their results are consistent with the findings of this meta-analysis. However, the processes by which bariatric surgery improves endothelium damage biomarkers are mostly unknown. It is likely that the key mechanism responsible for the decrease of these indicators is the reduction of adipose tissue [51].

It is difficult to explain why there was no association between the changes in BMI and absolute difference in MCP-1 levels. The reason might be that although it is the most widely used indicator of obesity status in clinical settings and population health research, BMI is not the optimal measure for obesity. Since BMI is an indirect measure of obesity, it does not account for the location of adipose tissue (subcutaneous vs. visceral fat) differentiate between fat mass or lean mass (muscle mass, bone density etc.), or account for variation in body composition [52]. This might be the answer as to why no association between the changes in BMI and absolute difference in MCP-1 levels could be found. However, various mechanisms other than decreased fat tissue mass, such as decreased inflammation, decreased nutrient absorption, lower energy intake, or decreased need for the liver to detoxify ingested drugs, might have an impact on circulating MCP-1 levels as well [53].

In line with previous study, we showed that both LSG and RYGB improve the obesity and inflammatory conditions of patients, However, a gastric bypass was found to be more beneficial as compared to gastrectomy [54].

Most pro-inflammatory cytokines began to decrease early after surgery and continued to decline in the medium- and long-term. The current study found that MCP-1 decreased with weight loss and that this drop was consistent in long-term follow-up. In this sense, metabolic improvement seems to be an early change after bariatric surgery that may favor obesity-induced inflammation resolution [55].

The decrease of MCP-1 after bariatric surgery as an indication of anti-inflammatory effect might offer subsequent protection from obesity-related comorbidities such as insulin resistance, ACVD, and maybe some types of cancer, which are all associated with obesity.

This meta-analysis has certain limitations: some studies did not have a control group, had small patient groups, and were not randomized; however, the results were still strong following the leave-one-out sensitivity analysis. Second, we were unable to account for the impact of different bariatric surgery approaches, which could result in a significantly higher or reduced response.

## 5. Conclusions

Bariatric surgery significantly decreases MCP-1 concentration, but there was no association between the changes in BMI and absolute difference in MCP-1 levels before and after the surgery. However, a linear relationship between the changes in MCP-1 and the length of follow-up has been shown. A reduction in circulating levels of MCP-1 could be regarded as a potential factor in explaining the positive impact of bariatric intervention on cardiometabolic outcomes beyond weight loss.

This systematic review and meta-analysis was not registered.

## Figures and Tables

**Table 1:** Characteristics of studies measuring MCP-1.

Study, Year, Country	Study Design	Follow-Up	Type of Surgery	Clinical Outcome		Patients	No. of Patients
				MCP-1 Level Change	% BMI Change		
Salman 2021 [24]	Prospective study	12 months	LSG	Unchanged	−10.22 kg/m^2^	Obese non-diabetic patients	61
Rizk 2021 [25]	Prospective longitudinal research	3 months	LSG	Significant reduction	−15.96 kg/m^2^	Class III obesity subjects	24
Morales 2021 [26]	Prospective observational study	12 months	LSG, also known as RYGB	Significant reduction	−14.20 kg/m^2^	Obese patients with CKD	30
Yan 2021 a [27] Yan 2021 b [27]	Prospective randomized study	1 month 3 months 6 months 12 months	RYGB LSG	Significant reduction Significant reduction after 6, also known as 12 months	−8.30 kg/m^2^ (after 12 months) −8.80 kg/m^2^ (after 12 months)	Overweight and obese patients with BMI > 28 kg/m^2^ and type-2 diabetes	77 80
Bratti 2021 [28]	Prospective study	6 months	LSG, also known as RYGB	Unchanged	−15.47 kg/m^2^	Severe obesity	40
Salman 2020 [29]	Prospective study	12 months	OAGB	Significant increase in MCP-1 level	−10.07 kg/m^2^	Obese patients	62
Lambert 2018 [30]	Prospective study	1–2 months 12 months	BPD, also known as RYGB	Unchanged Significant reduction	−11.8 kg/m^2^	Obese patients	109
Alsharidah 2018 [31]	Prospective study	3 months	Mixed	Significant reduction	−6.5 kg/m^2^	Patients with NAFLD and obesity	51
Yadav 2017 [32]	Prospective study	6 months 12 months	RYGB	Significant reduction	−17 kg/m^2^ (after 12 months)	Obese patients	37
van der Wielen 2017 [33]	Prospective study	12 months	Gastroplication	Unchanged	−6.4 kg/m^2^	Morbidly obese patients	10
Sams 2016 a [34] Sams 2016 b [34]	Case-control study	2 weeks 6 months 2 weeks 6 months	RYGB LAGB	Unchanged	−12.7 kg/m^2^ −4 kg/m^2^	Obese patients	8 2
Kelly 2016 a Kelly 2016 b [35]	Longitudinal cohorts Longitudinal cohorts	6 months 12 months 6 months 12 months	LSG, also known as RYGB RYGB	Unchanged	−16.63 kg/m^2^ −20.9 kg/m^2^	Obese adolescents	39 13
Immonen 2014 a Immonen 2014 b [36]	Prospective study	6 months 6 months	LSG, also known as RYGB	Unchanged	−10 kg/m^2^ −9.8 kg/m^2^	Diabetic obese patients Non-diabetic obese patients	9 14
Gumbau 2014 [37]	Prospective study	1 day 5 days 1 month 6 months 12 months	LSG	Significant reduction after 12 months	−15.34 kg/m^2^ (after 12 months)	Morbidly obese	20
Bachmayer 2013 [5]	Prospective observational study	10 ± 6 months	Mixed	Unchanged	−13.4 kg/m^2^	Obese patients	21
Brinklov Thomsen 2013 a Brinklov Thomsen 2013 b [38]	Prospective cohort study	1 week 3 months 12 months 1 week 3 months 12 months	RYGB	Significant reduction	−30.52 kg/m^2^ −29.86 kg/m^2^	Obese patients without diabetes Obese patients with diabetes	10 10
Lima 2013 [39]	Prospective study	1 month 6 months 12 months	RYGB	Significant reduction	−16.4 kg/m^2^	Premenopausal women with metabolic syndrome and grade III obesity	10
Monte 2012 [40]	Prospective study	6 months	RYGB	Significant reduction	−11.7 kg/m^2^	Obese diabetic patients	15
Dalmas 2011 [41]	Case-control study	3 months 6 months 12 months	RYGB	Significant reduction after 3 and 12 months	−13.4 kg/m^2^	Obese women	51
Schaller 2009 [42]	Prospective observational study	18 ± 3 months	RYGB, also known as LGB	Significant reduction	−13.1 kg/m^2^	Morbidly obese patients	31
Hempen 2009 [43]	Case-control study	17.4 months	RYGB	Significant reduction	−13.2 kg/m^2^	Obese patients	17
Swarbrick 2008 [44]	Prospective study	12 months	RYGB	Unchanged	−14.8 kg/m^2^	Obese women	19
Catalán 2007 [1]	Case-control study	13 months	RYGB	Unchanged	−15.8 kg/m^2^	Obese women	14
Fontana 2007 [45]	Case-control study	12 months	RYGB	Unchanged	−18.7 kg/m^2^	Women with class III obesity	6
Schernthaner 2006 [46]	Prospective study	26.6 ± 11.5 months	VBG	Significant reduction	−12 kg/m^2^	Obese patients	37

LGB: laparoscopic gastric banding, LSG; laparoscopic sleeve gastrectomy, RYGB: Roux-en-Y gastric bypass, VBG: vertical banded gastroplasty surgery.

**Table 2:** Quality assessment of the included studies in accordance with the Newcastle–Ottawa scale (for observational studies) and Cochrane Collaboration’s tool (for randomized controlled trial).

Study	Selection					Comparability	Outcome		
	Representativeness of the Exposed Cohort	Selection of the Non-Exposed Cohort		Ascertainment of Exposure	Demonstration That Outcome of Interest Was Not Present at Start of Study	Comparability of Cohorts on the Basis of the Design or Analysis	Assessment of Outcome	Was Follow-Up Long Enough for Outcomes to Occur	Adequacy of Follow-Up of Cohorts
Salman 2021 [24]	*	-		*	*	-	*	*	*
Rizk 2021 [25]	*	*		*	*	-	*	-	-
Morales 2021 [26]	*	-		*	*	-	*	*	*
Yan 2021 [27]	*	-		*	*	-	*	*	*
Bratti 2021 [28]	*	*		*	*	*	*	*	*
Salman 2020 [29]	*	-		*	*	-	*	*	*
Lambert 2018 [30]	*	*		*	*	*	*	*	*
Alsharidah 2018 [31]	*	-		*	*	-	*	-	-
Yadav 2017 [32]	*	-		*	*	-	*	*	*
van der Wielen 2017 [33]	*	-		*	*	-	*	*	*
Sams 2016 [34]	*	-		*	*	-	*	*	*
Kelly 2016 [35]	*	-		*	*	-	*	*	*
Immonen 2014 [36]	*	*		*	*	*	*	*	*
Gumbau 2014 [37]	*	-		*	*	-	*	*	*
Bachmayer 2013 [5]	*	-		*	*	-	*	*	*
Thomsen 2013 [38]	*	-		*	*	-	*	*	*
Monte 2012 [40]	*	-		*	*	-	*	*	*
Dalmas 2011 [41]	*	*		*	*	*	*	*	*
Schaller 2009 [42]	*	-		*	*	-	*	*	*
Hempen 2009 [43]	*	-		*	*	-	*	*	*
Swarbrick 2008 [44]	*	-		*	*	-	*	*	*
Catalán 2007 [1]	*	-		*	*	-	*	*	*
Fontana 2007 [45]	*	*		*	*	*	*	*	*
Schernthaner 2006 [46]	*	*		*	*	*	*	*	*
	**Selection bias**				**Performance bias**	**detection bias**	**attrition bias**	**Reporting bias**	**other bias**
	Random sequence generation		Allocation concealment						
Lima 2013 [39]	Unclear		high		low	Unclear	low	low	low

## Data Availability

Not applicable.
